# Editorial of Special Issue “Roles of Inflammasomes and Methyltransferases in Inflammation”

**DOI:** 10.3390/ijms231810283

**Published:** 2022-09-07

**Authors:** Young-Su Yi, Miyong Yun

**Affiliations:** 1 Department of Life Sciences, Kyonggi University, Suwon 16227, Korea; 2 Department of Bioindustry and Bioresource Engineering, Sejong University, Seoul 05006, Korea

Inflammation is the first line of defense against pathogens and cellular dangers [1]. An inflammatory response consists of two successive steps: priming and triggering. Priming is the preparation step for inflammatory responses by upregulating the expression of various inflammatory molecules, while triggering is the activation step for inflammatory responses, and the key event of the triggering step is the activation of inflammasomes, intracellular multiprotein complexes that provide the platform for inflammatory responses [2,3]. Previous studies have demonstrated that inflammasomes play critical roles in inflammatory responses and diseases [4,5,6,7]; however, their roles are not yet fully understood.

Post-translational modifications (PTMs) are a critical determinant of various biological functions, including inflammation [8,9]. PTMs also occur in inflammasome components, resulting in the regulation of inflammasome functions [10,11]. Methylation is one of the PTMs through which methyl groups are added to biological molecules, leading to the epigenetic modification and functional regulation of these molecules. Therefore, methylation is critical for cellular homeostasis, and the dysregulation of methylation can cause various pathological conditions. In this context, methyltransferases may play key roles in inflammasome functions and inflammasome-mediated inflammatory responses.

This Special Issue invited original research, reviews, and perspectives with a focus on, but not limited to, the mechanisms of inflammasome regulation, the role of inflammasomes in inflammatory responses and disease, functional interplays between inflammasomes and methyltransferases, the identification and validation of novel molecules regulating the functions of inflammasomes and methyltransferases, and potential inflammasome- or methyltransferase-targeted therapeutics.

The research article by Lima et al. explored the role of Natterin, a potent pro-inflammatory fish molecule that can induce NLRP6 and NLRC4 inflammasome-activated neutrophilia, whether it activates mitochondrial damage, and also whether mitochondrial damage-mediated self-DNA leaks into cytosol can activate the cGAS and STING pathways in triggering the innate immune response. This study demonstrated that Natterin induced inflammation and mitochondrial damage, leading to the self-DNA leaks into the cytosol and that cGAS and STING are critical sensors of the self-DNA to regulate Natterin-activated innate immune response [12].

The research article by Sun et al. investigated the functional regulation of RIP2 (receptor-interacting serine/threonine kinase 2) mediated by transcription factors binding to a RIP2 promoter region in the immune response against avian pathogenic *E. coli* (APEC) infection. A previous study showed that the RIP2-mediated signaling pathway played a pivotal role in the immune response against APEC infection, and this study further demonstrated that the transcriptional regulation of RIP2 by transcription factor NFIB plays a key role in cellular immune and inflammatory response against APEC infection [13].

Another research article by the same research group further reported new microRNAs (miRNAs) that are potentially involved in the modulation of the APEC infection-induced inflammatory response. This study identified gga-miR-445-5p, which is differentially regulated by RIP2, and demonstrated that gga-miR-445-5p played a critical role in the inflammatory response against APEC infection via targeting IRF2 to regulate type I interferons expression [14].

The review article by Yi highlighted the studies investigating the roles of the caspase-11 non-canonical inflammasome in inflammatory liver diseases, including non-alcoholic fatty liver disease (NAFLD), non-alcoholic steatohepatitis (NASH), and inflammatory liver injuries, and the underlying molecular/cellular mechanisms. The caspase-11 non-canonical inflammasome is activated by sensing intracellular lipopolysaccharide (LPS), leading to the inflammatory form of cell death, known as pyroptosis, and the massive secretion of pro-inflammatory cytokines, which indicates the role of caspase-11 non-canonical-inflammasome-mediated inflammatory response in “non-sterile inflammation” [2,3]. The development of NAFLD and NASH is caused by an excessive accumulation of fat in the livers, and a majority of inflammatory liver injuries are caused by dander signals and stress in the livers, suggesting that these inflammatory liver diseases are induced in “sterile inflammation” conditions, which are not associated with LPS or Gram-negative bacterial infection. Interestingly, the studies discussed in this review demonstrated that caspase-11 non-canonical inflammasome also plays a pro-inflammatory role in the pathogenesis of these inflammatory liver diseases [15]. This review provides new knowledge of caspase-11 non-canonical inflammasome as a key regulator of inflammatory liver diseases in a sterile inflammatory condition and also suggests insights into the development of novel therapeutics to prevent and treat inflammatory liver diseases via targeting caspase-11 non-canonical inflammasome.

In conclusion, this Special Issue highlights the regulatory roles of inflammasomes and inflammasome-related molecules in inflammatory responses and diseases and the underlying molecular and cellular mechanisms. However, the absence of a study demonstrating the regulatory roles of methyltransferases and the functional interplay between methyltransferases and inflammasomes in inflammatory responses and diseases is a limitation of this Special Issue. We hope this Special Issue gains the interest of the scientific society to contribute to future studies exploring new roles of inflammasomes and methyltransferases in inflammatory responses and human diseases and sheds light on developing new effective and safe therapeutics to prevent and treat human inflammatory and infectious diseases via targeting inflammasomes and methyltransferases.
